# Rod-Driven OFF Pathway Responses in the Distal Retina: Dark-Adapted Flicker Electroretinogram in Mouse

**DOI:** 10.1371/journal.pone.0043856

**Published:** 2012-08-24

**Authors:** Bo Lei

**Affiliations:** 1 Department of Ophthalmology, the First Affiliated Hospital of Chongqing Medical University, Chongqing Key Laboratory of Ophthalmology, Chongqing Eye Institute, Chongqing, China; 2 Department of Ophthalmology, Department of Veterinary Medicine and Surgery, University of Missouri, Columbia, Missouri, United States of America; Dalhousie University, Canada

## Abstract

**Purpose:**

The rodent retina does not exhibit a positive OFF-response in the electroretinogram (ERG), which makes it difficult to evaluate its OFF-pathway functions *in vivo*. We studied the rod-driven OFF pathway responses by using a dark-adapted 10-Hz flicker ERG procedure in mouse.

**Materials and Methods:**

Conventional ERGs and 10-Hz dark-adapted flicker ERGs were obtained in wild-type mice (*C57BL/6*), in mice with pure rod (*cpfl1*) or pure cone (*rho^−/−^*) function, and in *nob1* mice which have a selective ON-pathway defect. To isolate the response from ON or OFF pathway, glutamate analogs 2-amino-4-phosphobutyric acid (APB, an ON pathway blocker) and cis-2, 3-piperidine-dicarboxylic acid (PDA, an OFF pathway blocker), were injected intravitreally.

**Results:**

The amplitude-intensity profile of the dark-adapted 10-Hz flicker ERG in the wild-type mice exhibits two peaks at middle and high light intensities. The two peaks represent rod- and cone-driven responses respectively. In APB-treated *C57BL/6* mice and in *nob1* mice, the dark-adapted ERG b-waves were absent. However, both rod- and cone-driven OFF pathway responses were evident with flicker ERG recording. At middle light intensities that activate only rod system, the flicker ERG responses in saline-injected *nob1* mice were similar to those in APB-injected *cpfl1* mice and wild-type mice. These responses are sensitive to PDA. The amplitudes of these rod-driven OFF pathway responses were approximately 20% of the total rod-driven flicker ERG responses.

**Conclusion:**

We demonstrate that the rod-OFF bipolar cell pathway is functional in the outer retina. The dark-adapted flicker ERG is practical for the evaluation of rod- and cone-driven responses, and the residual OFF pathway signals in subjects with ON pathway defects.

## Introduction

**Figure 1:**
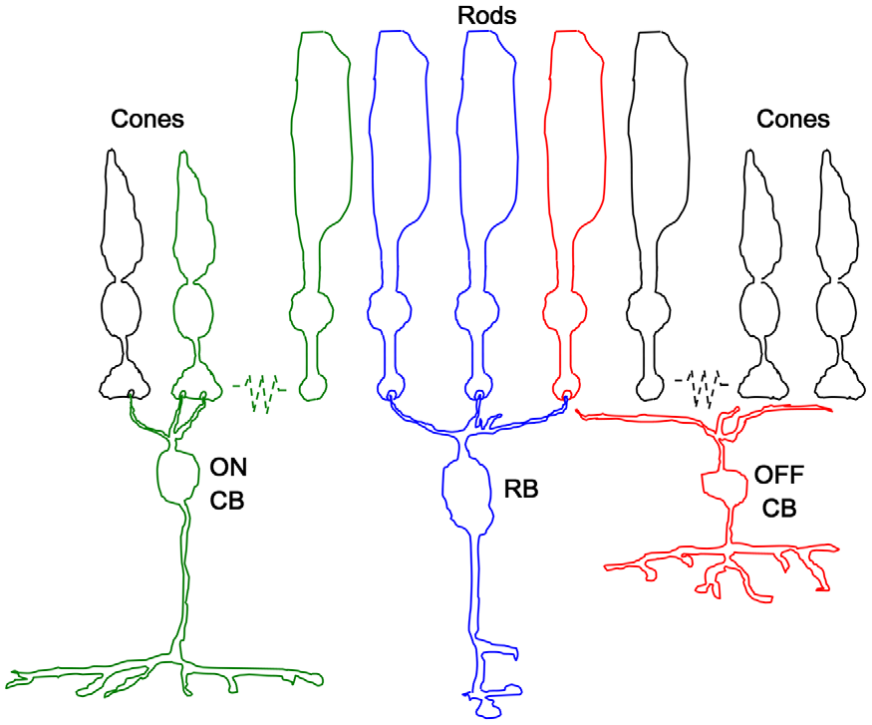
Schematic diagram of retinal rod pathways. The primary rod pathway (in blue) is from rods → rod bipolar cells. The second rod pathway (in green) is from rods → cones (through gap junctions) → ON and OFF cone bipolar cells. The third rod pathway (in red) is a direct connection between rods and OFF bipolar cells.

Two traditional rod pathways are known to exist in mammals [1], [2], [3]. The primary pathway for rod signals is transmission from rods → rod bipolar cells → AII amacrine cells → cone ON and OFF bipolar cells → ganglion cells. The second pathway for rod signals is from rods → cones (through gap junctions) → ON and OFF cone bipolar cells → ganglion cells (Fig. 1). Recent studies reveal the existence of a third rod pathway: a direct connection between rods and OFF cone bipolar cells [4], [5], [6], [7], [8], [9], [10]. This rod pathway appears to be a common feature of the mammalian retina [11], [12], [13], [14]. Ganglion cell responses mediated by this pathway have been documented in detail *in vitro* [14]. However, no study has been conducted to investigate the function of this newly discovered OFF pathway in electroretinogram (ERG), an objective and reliable method for evaluating the function of the outer retina in living animal.

To assess the third rod pathway function, two prerequisites must be satisfied. First, the responses must be triggered by the rods; secondly, the OFF pathway (including the third and part of the second rod pathways involving cone OFF bipolar cell) responses must be distinguishable from those generated from the ON pathway. Unfortunately, the OFF pathway responses in rodent are difficult to capture [15], [16], [17] and only small cone-driven OFF pathway responses have been observed in mouse [18]. The mouse dark-adapted ERG is dominated by ON bipolar cell responses of the first rod pathway [19]. ON bipolar cells responses can be excluded from ERG signals by using an ON channel blocker [16], [20], [21] in wild-type mice, or using mouse models with selective ON channel defects [22], [23], [24]. However, the remaining OFF pathway responses are overwhelmed by a huge negative photoreceptor ERG a-wave combined with the slow PIII component. A prolonged stimulation and other light-adapted ERG techniques have been applied to isolate the OFF pathway signals from the ON pathway responses [20], [25], [26]. However, the steady background light used in these procedures suppresses rod system function.

**Figure 2:**
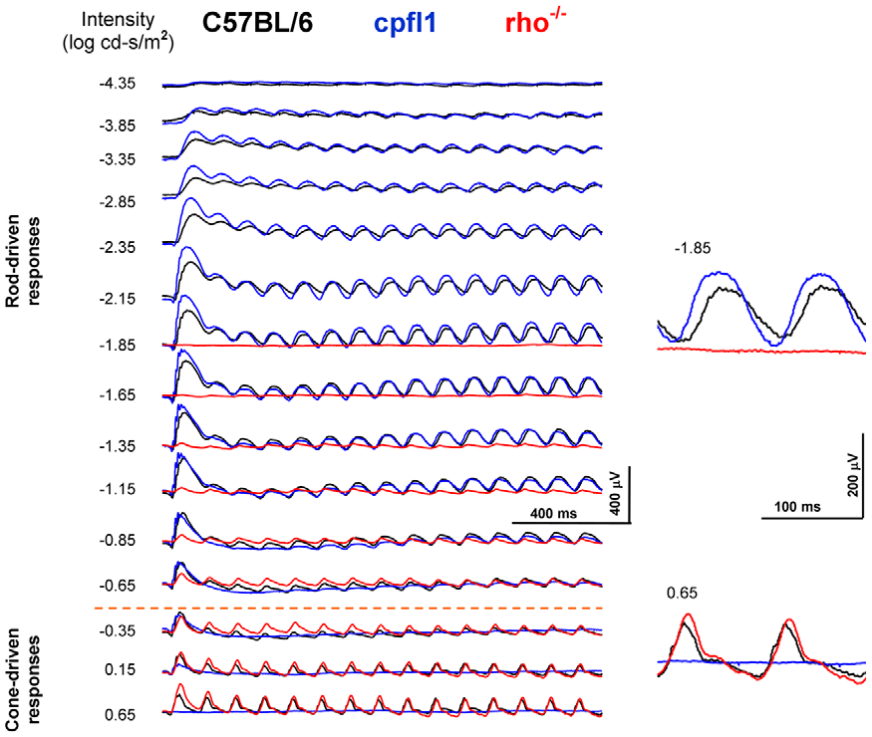
Dark-adapted10-Hz flicker ERGs elicited with a series of light intensity in *C57BL/6* (in black), *cpfl1* (in blue) and *rho^−/−^* (in red) mice. In the *C57BL/6* mouse, flicker ERGs can be divided into two phases. The first phase is from −3.85 to −0.65 log cd-s/m^2^, the waveforms appear sine wave in shape and the latencies were longer. The second phase is from −0.35 to 0.65 log cd-s/m^2^, the waveforms appear triangular wave and the latencies are shorter. In the *cpfl1* mouse, the responses of the first phase still exist, while the second phase responses are absent. In the *rho^−/−^* mouse, the first phase responses are absent, but the second phase responses remain. The results indicate that the first phase responses are rod-driven and the second phase responses are cone-driven. The dotted line indicates the light intensity break point where the flicker responses switch from rod-dominant to cone-dominant.

**Figure 3:**
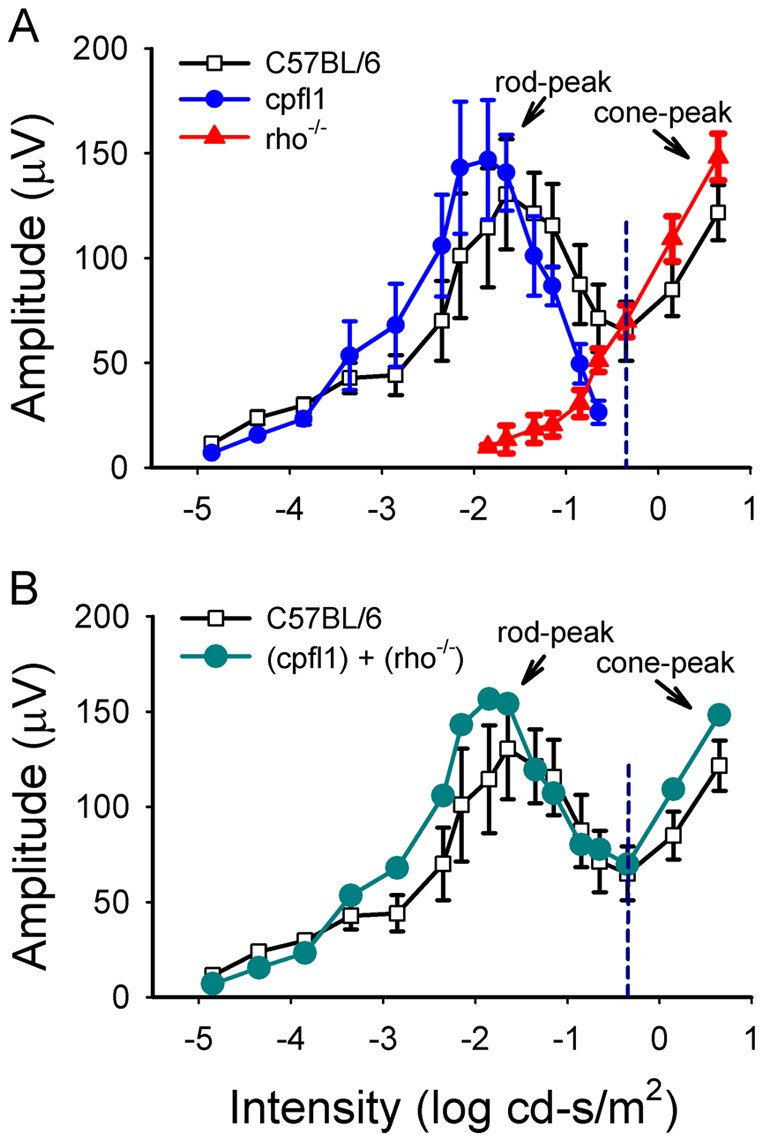
Dark-adapted 10-Hz flicker ERG response amplitude-intensity profiles in *C57BL/6*, *cpfl1* and *rho^−/−^* mice. There are two peaks in the wild-type mice, with the first representing rod-driven and the second representing cone-driven responses (Panel A). In *cpfl1* (n = 5) mice, the rod peak still exists while the cone peak is absent. In *rho^−/−^* (n = 4) mice the rod peak is absent, but the cone peak persists. The summation of the amplitude-intensity curves of the flicker ERG responses in *rho^−/−^* and *cpfl1* mice mimics the profile of the *C57BL/6* (n = 5) mice. (Panel B. Bars indicate the standard deviation.).

Here, we demonstrate that the OFF pathway responses, especially that generated from the third rod pathway, can be recorded with a dark-adapted 10-Hz flicker ERG protocol [27]. This study provides the first evidence *in vivo* that the function of the newly discovered third rod pathway can be detected with ERG. Its threshold is approximately 2.5 log units higher than that of the primary rod-ON pathway and about 1 log unit lower than that of the cone-driven OFF pathway responses. The amplitude of this pathway approximately accounts for 20% of the total rod-driven flicker responses.

**Figure 4:**
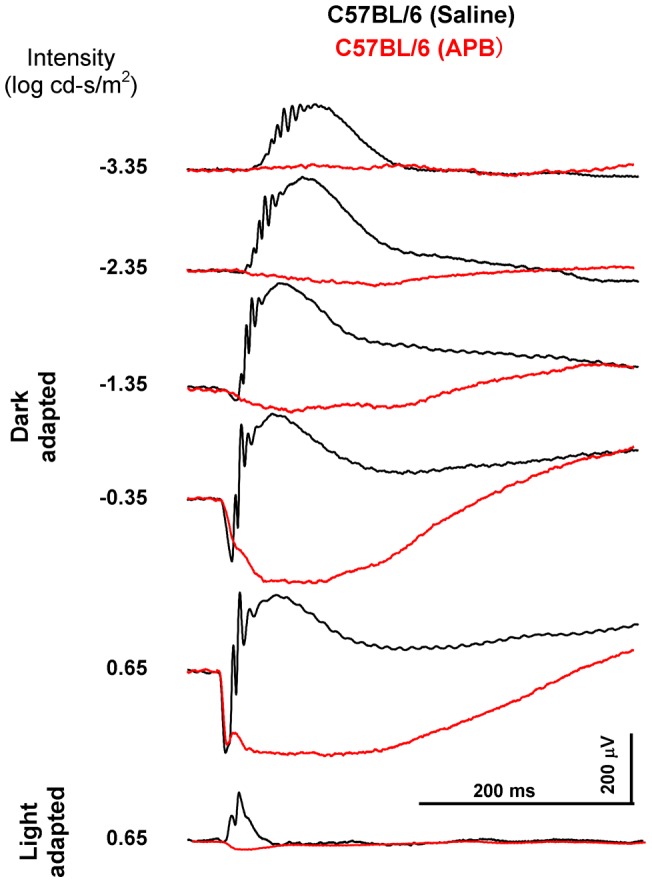
The effect of the ON-channel blocker APB on the dark- and light-adapted ERG in mouse. The black traces show ERGs of the saline-injected control eye and the red traces show ERGs of the APB-injected eye. In the APB-injected eye, the photoreceptor a-wave remains but the b-waves are absent. Loss of the b-waves indicates that APB blocks the signal transmission from the photoreceptors to the ON bipolar cells.

## Materials and Methods

All experiments were conducted in accordance with the Association for Research in Vision and Ophthalmology (ARVO) Statement for the Use of Animals in Ophthalmic and Vision Research. The experimental protocols were reviewed and approved (ID# 3713) by the Animal Care and Use Committee (ACUC) of the University of Missouri-Columbia.

**Figure 5:**
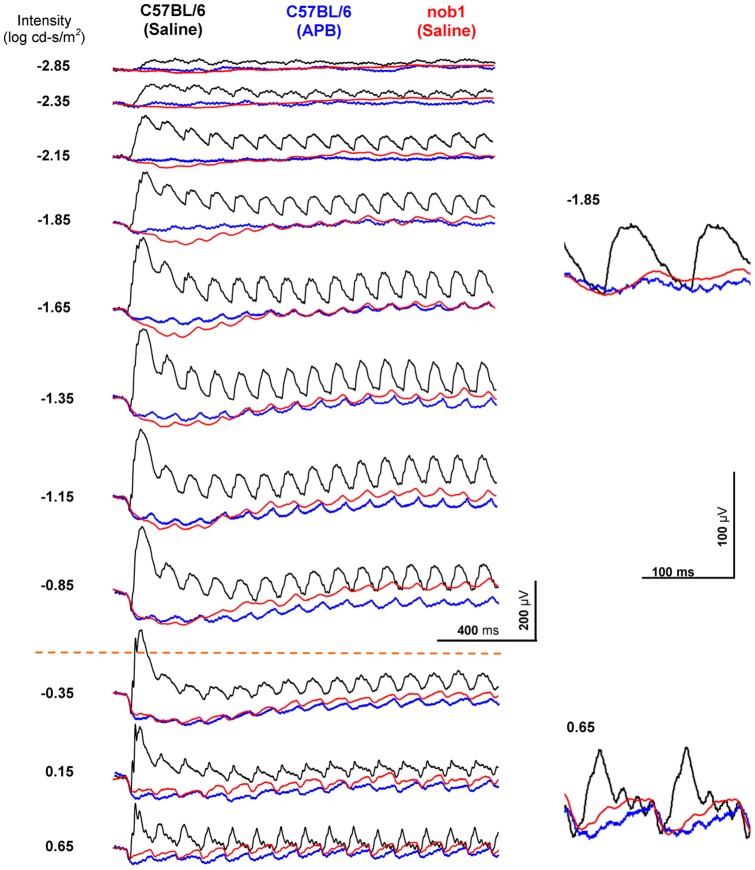
Dark-adapted 10-Hz flicker ERG OFF pathway responses in mice. The black traces show the ERGs from a saline-injected eye and the blue traces show the ERGs from the contralateral APB-injected eye of a *C57BL/6* mouse. The red traces show the flicker ERGs from a saline-injected eye of a *nob1* mouse. The saline-injected eye of the wild-type mouse presents normal rod- and cone-driven flicker responses. The APB-injected *C57BL/6* mouse eye and the saline-injected *nob1* mouse eye show similar ERGs. The flicker response amplitudes decrease and the thresholds increase. In the APB-injected *C57BL/6* mouse and in the saline-injected *nob1* mouse, residual flicker responses (∼20% of the control) persist at intensity levels where signals are rod- or cone-driven.

The wild-type *C57BL/6*, *cpfl1* (cone photoreceptor function loss 1, generously provided by Dr. Bo Chang), and *nob1* (no ERG b-wave 1, generously provided by Dr. Neal Peachey) mice were obtained from Jackson Laboratory (Bar Harbor, ME). The original functional pure cone [28], [29] rhodopsin knockout mice (*rho^−/−^*, generously provided by Peter Humphries). All of the mice have the same genetic background (*C57BL/6*). Because cone system function of the *rho^−/−^* mice starts to deteriorate at 7 weeks after birth [28], [29], all of the mice used in this study were 6 weeks old. Mice were housed under a 12 hour light/12 hour dark cycle with free access to food and water.

**Figure 6:**
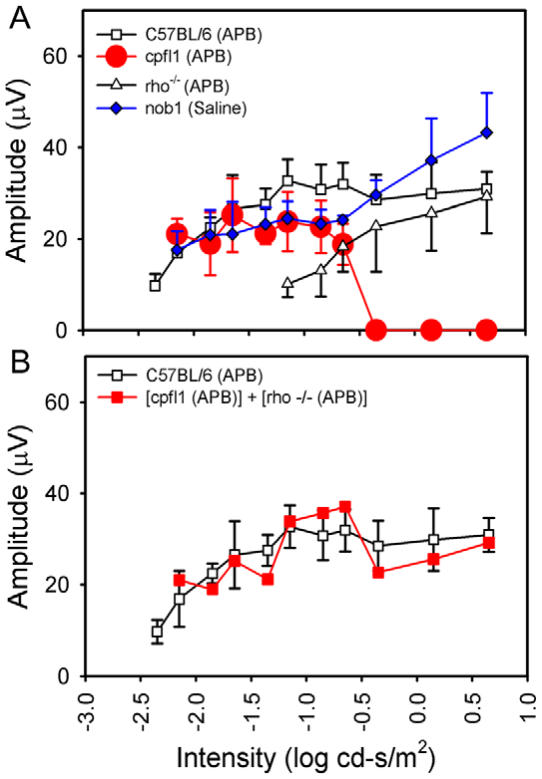
The amplitude-intensity curves of the rod- and cone-driven 10-Hz flicker ERG OFF pathway responses in mice. The OFF response threshold of the APB-treated *C57BL/6* mice (n = 5) is −2.35 log cd-s/m^2^ (Panel A). The amplitude increases with the intensity and reaches a plateau starting at −1.15 log cd-s/m^2^. The response threshold of the APB-injected *rho^−/−^* mice (n = 4) is −1.15 log cd-s/m^2^. The flicker ERG OFF pathway responses of the APB-injected *cpfl1* mice (n = 5) are seen at a light intensity range of −2.15 ∼ −0.65 log cd-s/m^2^. The threshold of the saline-injected *nob1* mice (n = 7) is comparable to that of the APB-injected wild-type mice. The summation of the flicker ERG amplitude-intensity curves of the APB-injected *rho^−/−^* and *cpfl1* mice resembles the profile of the APB-injected *C57BL/6* mice. ((Panel B. Bars indicate the standard deviation.).

Mouse ERGs were recorded using protocols modified from previous studies [30], [31]. Briefly, mice were dark adapted overnight and anesthetized with a mixture of ketamine (75 mg/kg intramuscularly) and xylazine (13.6 mg/kg intramuscularly). Pupils were dilated with 1% tropicamide, and a heating pad was used to keep the body temperature at 38°C. The corneal electrode was a gold wire loop; a reference electrode was placed on the forehead and a ground electrode was applied subcutaneously near the tail. Signals were amplified at 10,000 gain and bandpass filtered between 0.1 and 1000 Hz. The signals were digitized at 5.12 kHz for conventional ERG and at 2.06 kHz for 10-Hz flicker ERG recordings with a data acquisition device (National Instrument, Austin, TX). To increase the signal noise ratio, 3∼6 signals were averaged for conventional dark-adapted ERG; whereas 12∼16 signals were averaged for light-adapted responses and for the 10 Hz flicker ERGs, using a custom-compiled program (LabView 7.1, National Instrument, Austin, TX).

**Figure 7:**
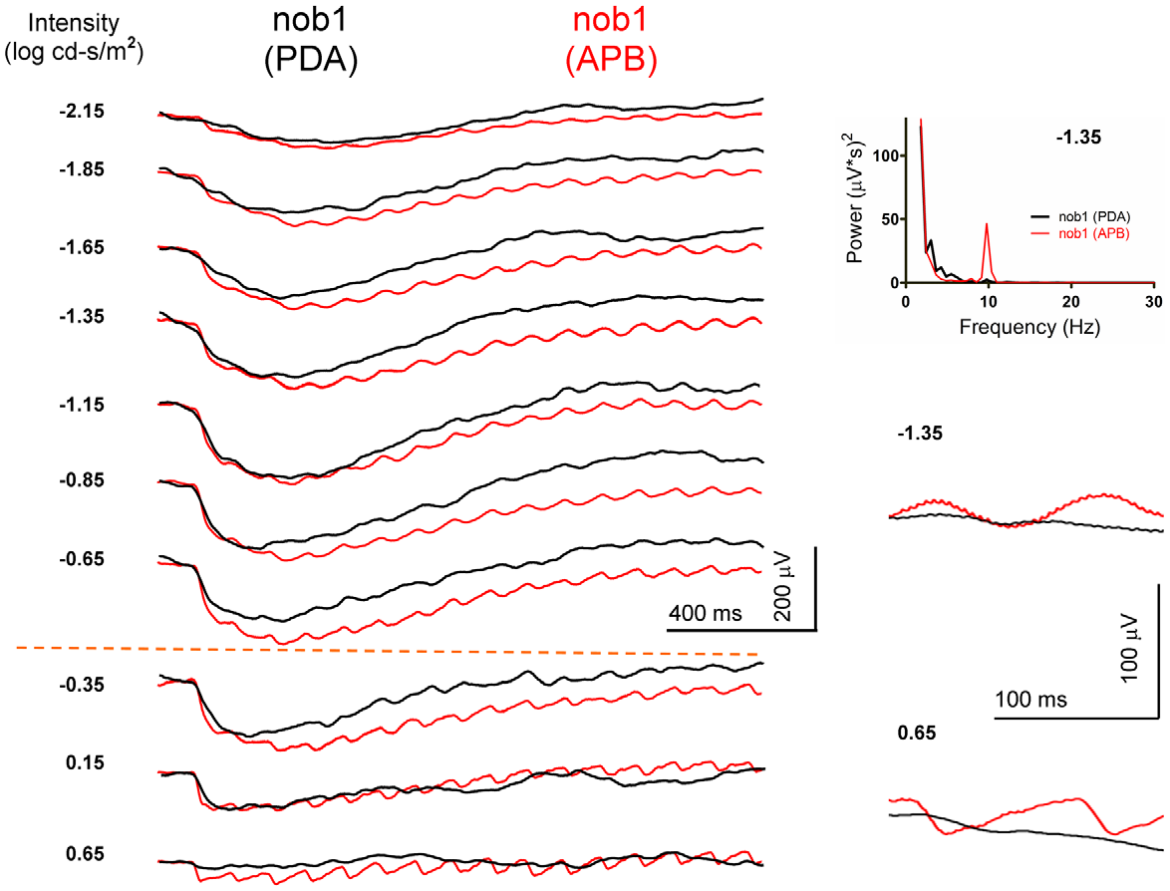
Effects of intravitreal injection of APB and PDA in a *nob1* mouse. Both eyes show negative waveforms after injections. In the PDA-injected eye, the rod- and cone-driven flicker ERG OFF pathway responses are reduced. However these responses are resistant to APB. Frequency spectra indicated that the 10 Hz components in the PDA injected eye are very small.

Ganzfeld was illuminated using white flash light provided by a Grass PS22 Xenon visual stimulator (Grass Instrument Inc. West Warwick, RI). The light flash had a duration of 10 µs, and the maximum intensity was 0.65 log cd-s/m^2^. A timer (Uniblitz, Rochester, NY) was used to control the frequency of the flash. In dark-adapted ERG recordings, the interstimulus interval (ISI) was at least 12 seconds for low intensities and more than 30 seconds for high intensities. In the light-adapted ERG recording, a background light of 30 cd/m^2^ was applied to suppress rod responses. For the 10-Hz flicker ERG recording, the interval between the two consecutive flash trains was 200 milliseconds. Stimulus light intensity was attenuated with neutral density filters (Kodak, Rochester, NY). Luminance was calibrated with an IL-1700 integrating radiometer/photometer (International Light, Newburyport, MA). ERG signals were analyzed off-line using custom-compiled programs developed in LabView 7.1 (National Instrument, Austin, TX). The amplitude of the flicker responses was defined as the difference between the trough and the peak. Because the flicker responses of the number 10∼12 wavelets were relatively stable through all the intensity levels, we used their average as the flicker amplitude.

**Figure 8:**
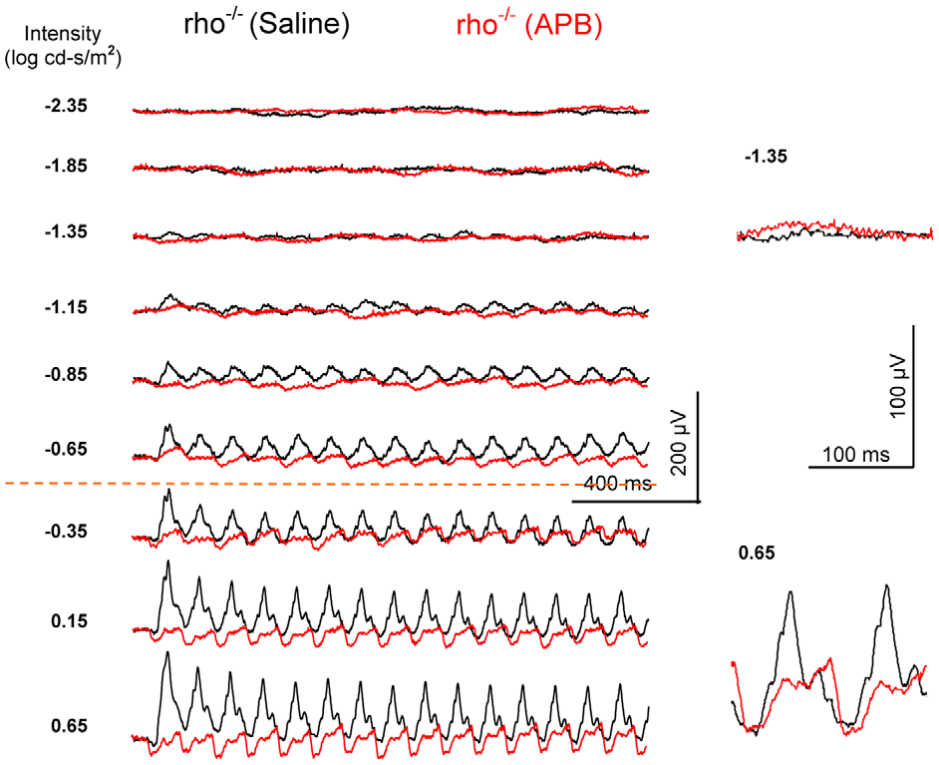
The effect of the ON-channel blocker APB on the light-adapted (background light 30 cd/m^2^) 10-Hz flicker ERG of a *rho^−/−^* mouse. The black traces show ERGs of the saline-injected eye and the red traces show ERGs of the APB-injected contralateral eye. When compared with the control eye, the threshold of flicker ERG responses in the APB-injected eye increases and the amplitudes decrease.

**Figure 9:**
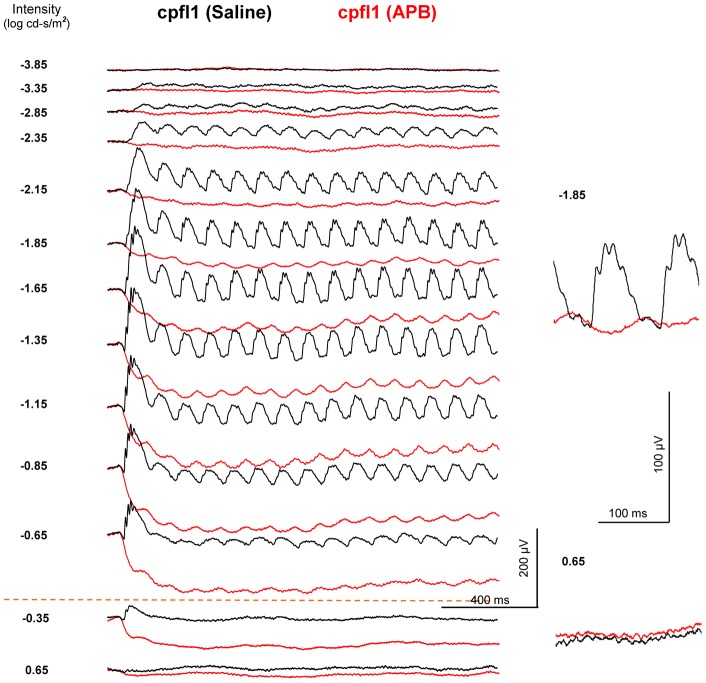
The effect of the ON-channel blocker APB on the dark-adapted 10-Hz flicker ERG of a *cpfl1* mouse. The black traces show ERGs of the saline-injected control eye and the red traces show ERGs of the APB-injected contralateral eye. The threshold of flicker ERG response increases and the amplitude decreases in the APB-injected eye. The dark-adapted 10-Hz flicker ERG responses exist at the medium intensities (−2.15 ∼ −0.65 log cd-s/m^2^), but are absent at high intensities.

The ON channel blocker2-amino-4-phosphonobutyric acid (APB) and the OFF channel blocker cis-2, 3-piperidine-dicarboxylic acid (PDA) were purchased from Sigma-Aldrich (St. Louis, MO). Solutions were filtered through 20-micro filters. The same volume (1.5 µl) of the drug solution and control vehicle saline was injected into the mouse eyes with Hamilton syringes. Based on our measurement from fresh eyes and data in the literature [32], the interior diameter of the eyeball (retinal surface) is approximately 2.8 mm and the lens diameter is approximately 2.1 mm. Vitreous volume of mice was estimated to be around 6.6 µl and the intravitreal drug concentrations were 8.2 mM for APB and 26.3 mM for PDA.

## Results

The conventional dark- and light-adapted ERGs of each of the 4 mouse strains are similar to previous reports. The dark- and light-adapted ERG waveforms and amplitudes of the wild-type *C57BL/6* mice, pure rod function *cpfl1* mice [30], [33], [34], pure cone function *rho^−/−^* mice [28], [29], [30], and selective ON pathway defect *nob1* mice [23], [24] were similar to those obtained in previous studies over a 6 log unit range of intensities (data not shown).

### The dark-adapted 10-Hz flicker ERGs

Dark-adapted 10-Hz flicker ERGs of wild-type *C57BL/6*, *cpfl1* and *rho^–/–^* mice are shown in figure 2 and the amplitude-intensity profiles of the three strains are shown in figure 3. In wild-type mice, the flicker threshold was −4.85 log cd-s/m^2^ (Figure 2A, 10 μV criteria). The amplitude of the flicker ERG increased and reached the first apex at −1.65 log cd-s/m^2^. As the intensity continuously increased, the amplitude decreased and reached a nadir at −0.35 log cd-s/m^2^. Then the flicker amplitude steadily increased again until 0.65 log cd-s/m^2^, the highest intensity available in our system. The flicker ERG waveforms were different morphologically below and above −0.35 log cd-s/m^2^: the ERGs of the first phase resembled sine waves with a longer latency; whereas the waveforms that formed the second phase were triangular with a shorter latency.

In *cpfl1* mice of pure rod system function, the flicker threshold was −4.65 log cd-s/m^2^ (Figures 2 and 3A, 10 μV criteria), which is comparable to that of the wild-type mice. The first increase phase still existed and peaked at stimulation intensity of −1.85 log cd-s/m^2^, which is similar to that of the wild-type mice. However, in contrast to the second increasing phase in *C57BL/6* mice, the response in the *cpfl1* mice continued to decrease at intensities higher than −0.35 log cd-s/m^2^. The flicker ERG waveforms of these mice were similar to those of wild-type mice recorded at the middle intensities, from −4.35 to −1.15 log cd-s/m^2^.

In *rho^−/−^* mice of pure cone functions, the flicker ERG response was not detectable at light intensities below −1.85 log cd-s/m^2^ (Figures 2 and 3A). At stimulation intensities from −0.35 to 0.65 log cd-s/m^2^, both the flicker waveforms and the amplitudes in the *rho^−/−^* mouse were similar to those of the wild-type mouse.

Because cone photoreceptors are not activated at flash intensity below −1.85 log cd-s/m^2^ (Figures 2 and 3A), the first increasing phase of the flicker ERG in wild-type mice must be driven by the rod system. When the flash intensity was higher than −1.85 log cd-s/m^2^, rod system functions was gradually suppressed by the increasing intensity of stimulation light and the ERG amplitude started to decrease. At the same time, the cone system became activated with the increasing light intensity. The trough at around −0.35 log cd-s/m^2^ (Figure 3A) indicates the break point where the cone system function start to be dominant. Beyond this intensity, no flicker responses were detected in pure rod *cpfl1* mice, indicating that rod system ERG responses were completed suppressed. Therefore the responses observed in wild-type mice at these high intensities were cone-driven. This rod-suppressing effect is similar to what observed in light-adapted ERG recordings using a steady background light. The cone-driven flicker responses elicited with the same light intensities were similar to those obtained in light-adapted condition (data not shown).

Figure 3B shows the summation of the amplitude-intensity curves of *rho^−/−^* and *cpfl1* mice. The summed rod- and cone-driven flicker response profile of these two strains mimics that of the wild-type mice. Thus the rod- and cone-driven responses are distinguishable with the dark-adapted 10-Hz flicker ERG. In the amplitude-intensity curves, the first peak represents the rod-driven responses and the second peak represents the cone-driven responses.

### The Flicker ERG OFF pathway responses

To isolate the ERG responses generated from the OFF pathway, APB was injected intravitreally to block the ON pathway of the rod and cone system [15], [16], [20], [35], [36]. About 90 minutes after the injection, the dark-adapted ERG b-wave was eliminated in the *C57BL/6* mouse, and a negative waveform was dominant at high intensities (Figure 4). The light-adapted b-wave was absent but the a-wave was evident. The ERG waveform of the APB-injected *C57BL/6* mice was identical to that of the *nob1* mice [23], [24]. Similar waveform has also been documented in patients with ON pathway defects and in monkeys after intravitreal APB injections [20], [21], [26], [36], [37].

In *C57BL/6* mice, the amplitudes of the dark-adapted 10-Hz flicker ERG of the APB-injected eyes were lower than the saline-injected contralateral eyes (Figures 5, 6A). Small flicker ERG responses were observed at middle intensities, starting at −2.35 ∼ −2.15 log cd-s/m^2^. The amplitude of the residual responses increased with flash intensity and reached a plateau at −1.15 log cd-s/m^2^ and was about 20% of the total flicker ERG responses of the control mice at −1.15 log cd-s/m^2^. Because the ON pathway was blocked by APB, these remaining flicker ERGs must be generated from the OFF pathway. The residual responses were recorded over a ∼3 log unit light intensity range (−2.35 ∼ 0.65 log cd-s/m^2^), which covers both the rod-driven (−1.85 ∼ −1.65 log cd-s/m^2^) and cone-driven (0.65 log cd-s/m^2^) flicker ERG responses observed in the wild-type mice (Figure 2).

The rod- and cone-driven OFF pathway responses were also observed in the saline-injected *nob1* mice (Figures 5, 6A), which show comparable waveforms and similar response-intensity curves as in APB-injected wild-type mouse. We further verified that the residual responses in *nob1* mice were generated from the OFF pathway by recording the flicker ERGs in seven PDA-injected and five APB-injected *nob1* mice (Figure 7). The negative a-waves remained and were comparable in these mice. However, the residual flicker responses were greatly reduced in PDA-injected eyes but remained in APB-injected eyes, indicating the signals are generated from the OFF pathway.

The threshold of the cone-mediated OFF pathway response was obtained from light-adapted flicker ERGs in intravitreal APB- and saline-injected *rho^−/−^* mouse eyes (Figure 8, n = 6). The amplitude of the flicker responses in APB-treated eyes decreased comparing with the saline-injected contralateral eyes. The threshold of the cone-driven OFF pathway responses was about −1.15 log cd-s/m^2^ (10 μV criteria) which represented a ∼0.7 log units increase over that of the saline-injected eyes.

### The OFF pathway responses mediated by the third rod pathway

The rod-driven OFF pathway responses in APB-injected *C57BL/6* mice and saline-injected *nob1* mice (Figure 5) may be mediated by the second (rod-cone gap junction) and/or the third (rod-OFF bipolar cell) rod pathways. To further clarify the cellular origin of the rod-mediated OFF pathway response, we recorded the flicker ERGs in five *cpfl1* mice after intravitreal APB injection (Figure 9). Peanut agglutinin lectin staining indicated that cone photoreceptors are morphologically normal in the 6-week-old *cpfl1* mice (data not shown). However, previous studies indicated that these cones are not functional and the cone system do not contribute to the ERG [30], [34], [38]. In the APB-injected *cpfl1* mouse eyes, the dark-adapted ERG b-waves were abolished and the a-waves remained. At middle intensities (−2.15 ∼ −0.65 log cd-s/m^2^), the rod-driven 10-Hz flicker ERG responses occurred similar to those observed in the APB-injected *C57BL/6* mice and saline-injected *nob1* mice (Figures 5, 6A). However, no cone-mediated responses were seen in the APB-injected *cpfl1* mice at higher intensities (above −0.65 log cd-s/m^2^). Because there are no contributions to the ERG from the cone system and thus the rod-cone gap junctions, and because the third rod pathway is resistant to APB [12], [14], the flicker ERG responses in these APB-injected *cpfl1* mice must be mediated through the third rod-pathway (i.e. the rod-OFF bipolar cell pathway).

Figure 6A shows the rod- and cone-driven OFF responses intensity-amplitude curves in mice. The flicker ERGs of the APB-injected *cpfl1* mice eyes (filled circles) represent the rod-driven OFF pathway responses. Figure 5B shows the summation of the OFF pathway amplitude-intensity profile of the APB-injected *rho^−/−^* and APB-injected *cpfl1* mice. The summed rod- and cone-driven flicker OFF pathway response curve resembles the OFF pathway responses of the APB-injected wild-type mice. The results suggest that both the rod- and cone-driven OFF pathway responses contribute to the OFF flicker responses in normal mice. The OFF responses elicited by intensities lower than −1.15 log cd-s/m^2^ were rod-driven, and the OFF responses elicited by intensities higher than −0.65 log cd-s/m^2^ were cone-driven. At light intensities between −1.15 and −0.65 log cd-s/m^2^, both the rod and cone photoreceptors contribute to the OFF pathway responses.

## Discussion

ERG is an objective and reliable assessment for the retina function *in vivo*. Because mice are extensively used as models of human diseases, it is imperative that retinal function of the mouse be fully understood. Studies in primates have demonstrated that the OFF pathway contribute to the light-adapted ERG a- and b-wave [20] and the flicker ERG responses [21], [39]. However, although the cone-driven OFF pathway responses were observed in *nob1* mice [18], mouse OFF pathway signals are still considered elusive [15], [16], [17]. Recently, a third rod pathway that directly connects the rods to the OFF cone bipolar cells has been demonstrated in the mouse, rat, rabbit and cat [4], [5], [6], [7], [8], [11], [12], [13], [14]. In this study, we demonstrated that mouse OFF pathway signals generated from this pathway are essential components in dark-adapted 10-Hz flicker ERG.

Our results indicate that, similar to the light-adapted flicker ERG in primates [21], [39], both ON- and OFF-pathway signals contribute to cone-driven, and more interestingly, the rod-driven flicker ERGs in mice. The rod-driven OFF pathway responses are integrated parts of the mouse ERG. In *nob1* and in the wild type mice injected with an ON-channel blocker APB, the flicker ERG OFF pathway responses were present over 3 log units from middle to high intensities. These responses are resistant to APB but sensitive to an OFF channel blocker PDA. These wavelets generated from the OFF pathway were neither observed in conventional dark- and light-adapted ERGs, nor in the light-adapted ERGs elicited with a long duration of light stimulation. The threshold of the OFF flicker response is about 2.5 log units higher than that of the composite flicker responses, which contain inputs from both the ON- and OFF-pathways.

We demonstrate that the recently discovered rod-OFF bipolar cell pathway is operational in the distal retina in *cpfl1* mice. The rod-driven flicker ERGs in the APB-treated *C57BL/6* mice and in saline-injected *nob1* mice could be mediated by both the second and the third rod pathways. However, the cone system are not functional in *cpfl1* mice and do not contribute to the ERGs [30], [34], [38]. Thus the rod-mediated flicker ERG responses in *cpfl1* mice are not activated through the rod-cone gap junctions. In addition, the primary rod pathway is not functional in the APB-injected *cpfl1* mice. Therefore the rod-driven flicker ERGs of the *cpfl1* mice represent the signals mediated through the rod-OFF bipolar cell pathway. The threshold of the flicker ERG rod-driven OFF pathway responses is about 1 log unit lower than that of the cone-driven OFF response (Figure 6A, −2.15 *vs*. −1.15 log cd-s/m^2^), which is coincidental to that demonstrated at the ganglion cell level *in vivo* [14]. The amplitude of the rod-driven OFF pathway responses accounts for about 20% of the total rod-mediated flicker ERG signals.

Our results suggest that the contribution from the third rod pathway is dominant in the rod-driven ERG OFF pathway responses. We observed similar thresholds and amplitudes of the rod-driven OFF pathway responses in the APB-injected *C57BL/6* and in *cpfl1* mice, suggesting the contributions of the second rod pathway to the ERG signals are minimal. In addition, the rod-driven ERG OFF pathway responses in the *nob1* and wild-type mice (Figure 6A. −2.15 and −2.35 log cd-s/m^2^ respectively) have similar thresholds as that in the *cpfl1* mice (−2.15 log cd-s/m^2^). Previous *in vitro* studies have shown that the second rod pathway–mediated OFF responses exhibit a 1 log unit lower threshold than those of the third rod pathway [14]. If the responses threshold observed at the ganglion cell level can be extended to the ERG signal and if the rod-driven OFF pathway responses contain significant input from the second rod pathway, the threshold of the flicker responses should have been about 1 log unit lower. However, the rapid decay of the flicker responses from −2.15 to −2.35 log cd-s/m^2^ in the *C57BL/6* mice (Figure 6) suggests that a light intensity lower than −2.35 log cd-s/m^2^ is unlikely to elicit significant OFF pathway responses.

The dominance of the third rod pathway over the second OFF pathway observed in this study is supported by other evidence. A recent study shows that the cone responses mediated thought the second rod pathway are much smaller than that in the rods themselves [40]. In addition, previous results indicate the rods connecting with the third pathway outnumber those with cone gap junctions. Therefore the contributions from the second rod pathway to the rod-driven flicker OFF ERG may be less significant.

The results of this study indicate that rod- and cone-mediated flicker responses are additive and can be differentiated by the dark-adapted flicker ERG recording. In addition, because the three rod pathways have different thresholds, this technique may be useful in isolating their responses, particularly the primary and the third rod pathway responses. Attempts have been made to assess functions of the primary and secondary rod pathway in humans and in mouse using the dark-adapted flicker ERG recording [41], [42], [43], [44]. A study showed distinct dark-adapted flicker ERG responses in a group of such patients with different genotypes [43]. These results imply that OFF pathway function is affected to different extents in these subjects. With a carefully designed flicker ERG protocol, it is possible to elucidate the mechanisms of the remaining visual function in individuals with selective ON pathway dysfunction.
